# Performance of large language models in the Chinese National Medical Licensing Examinations and Beyond: Scoping review

**DOI:** 10.1371/journal.pdig.0001773

**Published:** 2026-10-09

**Authors:** Hui Zong, Jiao Wang, Jiaxue Cha, Rongrong Wu, Shanshan Hu, Yan Zhao, Bairong Shen

**Affiliations:** 1 Joint Laboratory of Artificial Intelligence for Critical Care Medicine, Department of Critical Care Medicine and Institutes for Systems Genetics, Frontiers Science Center for Disease-related Molecular Network, West China Hospital, Sichuan University, Chengdu, China; 2 Centre for Medical Big Data and Artificial Intelligence, The First Affiliated Hospital (Southwest Hospital) of Army Medical University (Third Military Medical University), Chongqing, China; 3 Shanghai Key Laboratory of Signaling and Disease Research, School of Life Sciences and Technology, Tongji University, Shanghai, China; 4 The First Affiliated Hospital of Soochow University, Suzhou, China; 5 Department of Prevention Healthcare, The First Affiliated Hospital (Southwest Hospital) of Army Medical University (Third Military Medical University), Chongqing, China; Botho University, LESOTHO

## Abstract

Large language models (LLMs) such as GPT-4 and DeepSeek have shown potential in medical education and assessment. In China, the Chinese National Medical Licensing Examination (CNMLE) has become a critical benchmark for evaluating LLMs’ professional knowledge and reasoning ability in non-English contexts. This scoping review aims to synthesize research evaluating LLM performance on the CNMLE and related Chinese medical examinations, identifying performance trends, methodological gaps, and future directions. A scoping review was conducted in accordance with PRISMA-ScR guidelines. PubMed and Web of Science were searched on June 12, 2025, using terms related to LLMs and Chinese medical examinations. Studies were included if they evaluated any LLM on national-level Chinese medical exams and reported performance metrics. Two reviewers screened and extracted data. Study quality was assessed using a 12-item checklist covering dataset characteristics, LLM setup, and evaluation methods. Fisher’s exact test was used to assess differences in the passing rates of different LLMs. 14 studies were included, covering 51 evaluation records across 8 types of Chinese medical examinations, including CNMLE and specialty exams such as critical care, radiation oncology, and ultrasound medicine. Exam years ranged from 2017 to 2024, with a shift toward using recent exams. A total of 9 LLMs were evaluated, including GPT-3.5, GPT-4, GPT-4o, ERNIE, DeepSeek-R1, Qwen-72B, Baichuan2-7B, Baichuan2-13B and DISC-MedLLM. GPT-4o and DeepSeek-R1 achieved the highest scores of 552 (92.00%) and 523 (87.20%) in the 2024 CNMLE, respectively, both surpassing the passing threshold. In an exploratory comparison, GPT-4 achieved a higher pass rate than GPT-3.5 with a statistically significant difference, although inconsistencies in datasets, model transparency, and evaluation criteria persist across the included studies. LLMs show improving performance on Chinese medical exams, especially newer models. Nevertheless, future research should prioritize standardized benchmarks, multimodal capabilities, and transparent evaluation to ensure meaningful clinical relevance and educational value.

## Author summary

Large language models (LLMs) like GPT-4 and DeepSeek are emerging as powerful tools in medical education and assessment. In China, the Chinese National Medical Licensing Examination (CNMLE), a standardized exam used to certify physicians, has become a key benchmark for evaluating whether LLMs can understand and reason through real-world clinical content in a non-English setting. In this scoping review, we analyzed 14 peer-reviewed studies that evaluated the performance of 9 different LLMs, including both GPT series and Chinese open-source LLMs, on the CNMLE and seven other types of national medical examinations in China. We found that LLMs such as GPT-4o and DeepSeek-R1 achieved high scores on recent exams, in some cases surpassing the official passing threshold. However, studies varied widely in their methods, data transparency, and evaluation criteria, highlighting the need for more standardized benchmarks and testing protocols. Our findings offer a comprehensive overview of how LLMs are currently performing on Chinese medical exams and outline the key challenges and future directions for integrating these models into medical education and licensing systems. This research provides timely insights for developers, educators, and policymakers working at the intersection of AI, healthcare, and global medical training.

## Introduction

Large language models (LLMs) such as ChatGPT, DeepSeek, Qwen and others have rapidly emerged as transformative tools across a wide range of domains, from natural language understanding to professional-level reasoning and content generation [1–5]. In the medical field, the application of LLMs needs to undergo rigorous evaluation [6–9], one of the most common of which is the competency assessment in the medical licensing examinations [10,11]. This trend has garnered global attention, especially as LLMs are increasingly tested on real world national medical licensing examinations, which are traditionally reserved for human professionals [12]. Performance on standardized medical examinations primarily reflects the ability of LLMs within constrained testing environments and should not be considered equivalent to real-world clinical competence. Notably, recent studies have begun to investigate whether LLMs can succeed in such exams in languages beyond English, with the Chinese National Medical Licensing Examination (CNMLE) becoming a widely studied benchmark.

In China, the CNMLE serves as the official gateway for certifying licensed physicians and is known for its breadth, complexity, and alignment with clinical practice [13]. It poses a unique challenge for LLMs, not only due to linguistic and cultural factors, but also because of its structured design, large item banks, and strict passing criteria. A growing number of studies have evaluated LLMs on CNMLE and related Chinese medical examinations. For example, Zong et al. evaluated ChatGPT across five years (from 2017 to 2021) of the CNMLE, finding that it consistently failed to meet the passing threshold, though it demonstrated potential in select disciplines such as epidemiology and dermatology [14]. Luo et al. systematically compared GPT-3.5, GPT-4, and GPT-4o on the 2020 and 2021 CNMLE, reporting significant improvements in accuracy for GPT-4o, particularly in complex question formats and specific clinical units [15]. Wu et al. compared DeepSeek-R1 and GPT-4o on 2024 CNMLE with 600 questions, revealing that both models achieved high accuracy and passed the exams [16]. Moreover, domain-specific evaluations have been conducted in fields such as radiation oncology [17], critical care [18], ultrasound medicine [19].

Although these studies provide an increasing amount of evidence, there are still several challenges. These studies vary substantially in terms of medical exam dataset, LLMs versions, prompt engineering strategies, and evaluation criteria. Some studies evaluate only a single model on a small set of questions, while others lack comparisons across multiple models. Moreover, there is no standardized benchmark dataset or consistent evaluation methodology across studies. The question of whether LLMs can consistently meet official pass thresholds remains open, as performance varies depending on model architecture, prompt design, and exam content.

While several prior studies have synthesized LLM performance in medical licensing examinations from a global perspective, these reviews primarily focused on overall benchmark performance across multiple countries and examination systems. For example, Liu et al. conducted a systematic review and meta-analysis of ChatGPT performance across worldwide medical licensing examinations [10], while our previous multinational study provided a broader platform-based analysis of LLM evaluation in global medical exams [12]. However, in these global syntheses, the CNMLE was included only as a limited component of multinational datasets and was not examined in depth. In particular, the unique linguistic, regulatory, and methodological characteristics of Chinese medical examinations remain insufficiently explored.

This scoping review focuses specifically on evaluations of LLMs on CNMLE, as well as a range of specialized Chinese medical examinations beyond the CNMLE, including disciplines such as radiation oncology, critical care, ultrasound medicine, pharmacy, and nursing, enabling a more detailed and context-aware synthesis. The novelty of this review lies not only in its focus on Chinese-language evaluation settings, but also in its examination of Chinese-native LLMs such as DeepSeek, Qwen, and Baichuan, which are increasingly relevant in localized medical AI ecosystems. Moreover, compared with prior global reviews that primarily summarized overall accuracy outcomes, this review systematically analyzes methodological heterogeneity across studies, including differences in exam datasets, prompt engineering strategies, model versions, evaluation settings, and benchmark construction. Given the rapid expansion of Chinese medical LLM research and the growing importance of multilingual evaluation in digital health, a CNMLE-focused synthesis provides insights that may not be fully captured through broader multinational analyses.

## Methods

This study follows the PRISMA-ScR (Preferred Reporting Items for Systematic Reviews and Meta-Analyses extension for Scoping Reviews) guidelines [20], which provide a structured framework for reporting the rationale, methods, and results of scoping reviews (S1 PRISMA Checklist).

### Data sources and search strategy

We conducted a comprehensive literature search on June 12, 2025, using two major academic databases: PubMed [21] and Web of Science (WOS) [22]. The goal was to identify original studies evaluating the performance of LLMs in Chinese medical examinations. To ensure completeness and relevance, we employed three categories of keywords. The first category targeted terminology related to generative AI or LLMs (e.g., large language model, ChatGPT, LLaMA, DeepSeek, Qwen). The second category included synonyms and variations of medical examinations (e.g., medical licensing exam, medical exam). The third category involved country name (e.g., China, Chinese). Boolean operators and truncations were appropriately applied to construct robust and database-specific search queries. The complete search strategies for the different databases are provided in S1 Appendix for transparency and reproducibility. All search results were exported and organized using Microsoft Excel (Microsoft Corp, USA) for subsequent eligibility screening and information management.

In addition to these databases, we also conducted searches in IEEE Xplore and Scopus on June 11, 2025. The IEEE Xplore search returned 2 records, which were determined to be irrelevant to the focus of this study. The Scopus search yielded no relevant results. We also expanded our search to include Chinese-language databases. Searches in CNKI and WANFANG were conducted on November 5, 2025. The CNKI search returned 2 records, none of which were relevant to this study. The WANFANG search returned 13 records, one of which was related, but it consisted of practice questions and had a limited sample size. Thus, it was excluded from the analysis.

### Inclusion and exclusion criteria

Two researchers with expertise in medical informatics (HZ and JC) collaboratively developed inclusion and exclusion criteria based on previous published studies [12,14] and the research objectives of this study.

Inclusion criteria were as follows: 1) The study evaluated LLM performance on Chinese medical licensing or equivalent standardized medical examinations using official question banks or national-level datasets. 2) The study applied at least one LLM. 3) The study was an original research article. 4) The study was published after the year 2022. Exclusion criteria included: 1) Studies using data unrelated to official question banks or national medical licensing examinations in China. 2) Studies that did not involve the use of any LLMs. 3) Studies categorized as preprints, patents, commentaries, or editorials. 4) Studies did not report specific performance metrics of the LLMs.

Two researchers (HZ and JC) independently reviewed all candidate studies to assess their eligibility. In cases of disagreement regarding inclusion, a third researcher (BS, a senior expert with over 20 years of experience in biomedical informatics) provided adjudication to reach consensus.

### Data extraction and management

To comprehensively evaluate the capabilities of LLMs on Chinese national medical examinations, we defined a structured data extraction framework based on our previous study [12]. Manual data extraction was conducted to ensure accuracy.

The following information was manually extracted from each included study: 1) Name of the medical examination. 2) Year of the medical examination. 3) Type of exam questions. 4) Number of questions. 5) The passing criteria. 6) Name of the LLM. 7) Version of the LLM. 8) Number of correctly answered questions. 9) Final score achieved by the LLM. 10) Accuracy rate of the LLM. 11) Whether the LLM passed the exam. 12) Data availability. Additionally, bibliographic metadata of the included studies, such as title, authors, publication year, and journal name were also collected. The passing threshold across included studies was standardized as 60% accuracy, which is the most commonly used criterion in CNMLE and related Chinese medical examinations.

Two reviewers (HZ and JW) independently extracted data from each included study using Microsoft Excel spreadsheets. Discrepancies in data extraction were resolved through discussion, and when necessary, a third researcher (BS) was consulted to reach agreement. This multi-reviewer approach was adopted to minimize bias and enhance data reliability.

### Quality appraisal

To evaluate the methodological quality and potential risk of bias of the included studies, we employed a customized 12-item appraisal checklist that integrates and adapts quality assessment frameworks from two previous studies. One prior study developed a structured tool to assess performance of ChatGPT on national medical licensing examinations across different countries [10], covering aspects such as question source, LLM version, and evaluation setup. From that checklist, we retained relevant items on dataset reporting and testing configuration but excluded items not applicable to non-interactive examination tasks (e.g., repeated inquiries, evaluator blinding, empathy assessment). The other study is our prior publication, which constructed a data model, including exam information, data processing, and model performance, to track and evaluate the performance of multiple LLMs on medical exams worldwide [12]. Based on above two frameworks, we built a 12-item checklist tailored to the context of LLMs on CNMLE and beyond medical examinations (S2 Appendix). This checklist was developed through expert consensus and reflects our prior experience in evaluating LLM performance on the CNMLE, ensuring both methodological rigor and contextual relevance. It should be noted that this checklist has not undergone formal validation. Accordingly, the quality ratings reported in this review should be interpreted as indicative rather than definitive. The checklist was organized into three domains: dataset characteristics (e.g., exam source, year, number of questions, data availability), LLMs testing setup (e.g., model version, prompt design, testing interface), and evaluation methods (e.g., metrics, statistical analysis, pass/fail criteria). Each item was evaluated as “Yes” or “No”, depending on whether the study provided sufficient information for that criterion. A “Yes” received one point, and the total score determined the quality category of the study: scores of 10 or above were classified as high quality, scores between 8 and 9 as moderate quality, and scores below 8 as low quality. Two reviewers (HZ and JW) independently assessed each study using this checklist, and disagreements were resolved through discussion with a third senior reviewer (BS) with extensive experience in medical artificial intelligence research.

### Statistical analysis

To assess differences in pass rates among LLMs, we conducted Fisher’s exact test to compare categorical outcomes. Fisher’s exact test was chosen because it is suitable for analyzing contingency tables with small or uneven sample sizes, which aligns with the distribution of evaluation records in this study. Statistical analyses were performed using the scipy.stats module from the SciPy library (version 1.7.1) in Python.

### Ethical considerations

This study was conducted using data entirely obtained from publicly accessible sources, without involving any human participants or animals. No confidential or personally identifiable patient information was accessed or analyzed. As such, in accordance with the ethical regulations of the authors’ affiliated institution (West China Hospital), this study did not necessitate formal review or approval by an ethics committee.

## Results

### Literature screening and selection

The systematic search yielded a total of 63 records (PubMed: 26; WOS: 37). After removing duplicates, 36 unique articles remained. In the first screening step, 9 articles were excluded based on publication type, including 5 preprints, 3 conference papers, and 1 patent. A content review of these studies confirmed their misalignment with this review scope, as none involved formal evaluation of LLMs on Chinese medical licensing examinations. The remaining 27 articles were then assessed by title and abstract. Of these, 13 were excluded due to irrelevance to the topic or the absence of reported performance metrics. Ultimately, 14 articles with 51 evaluation records met all eligibility criteria and were included in the final analysis. The full screening and selection process is shown in Fig 1.

**Fig 1:**
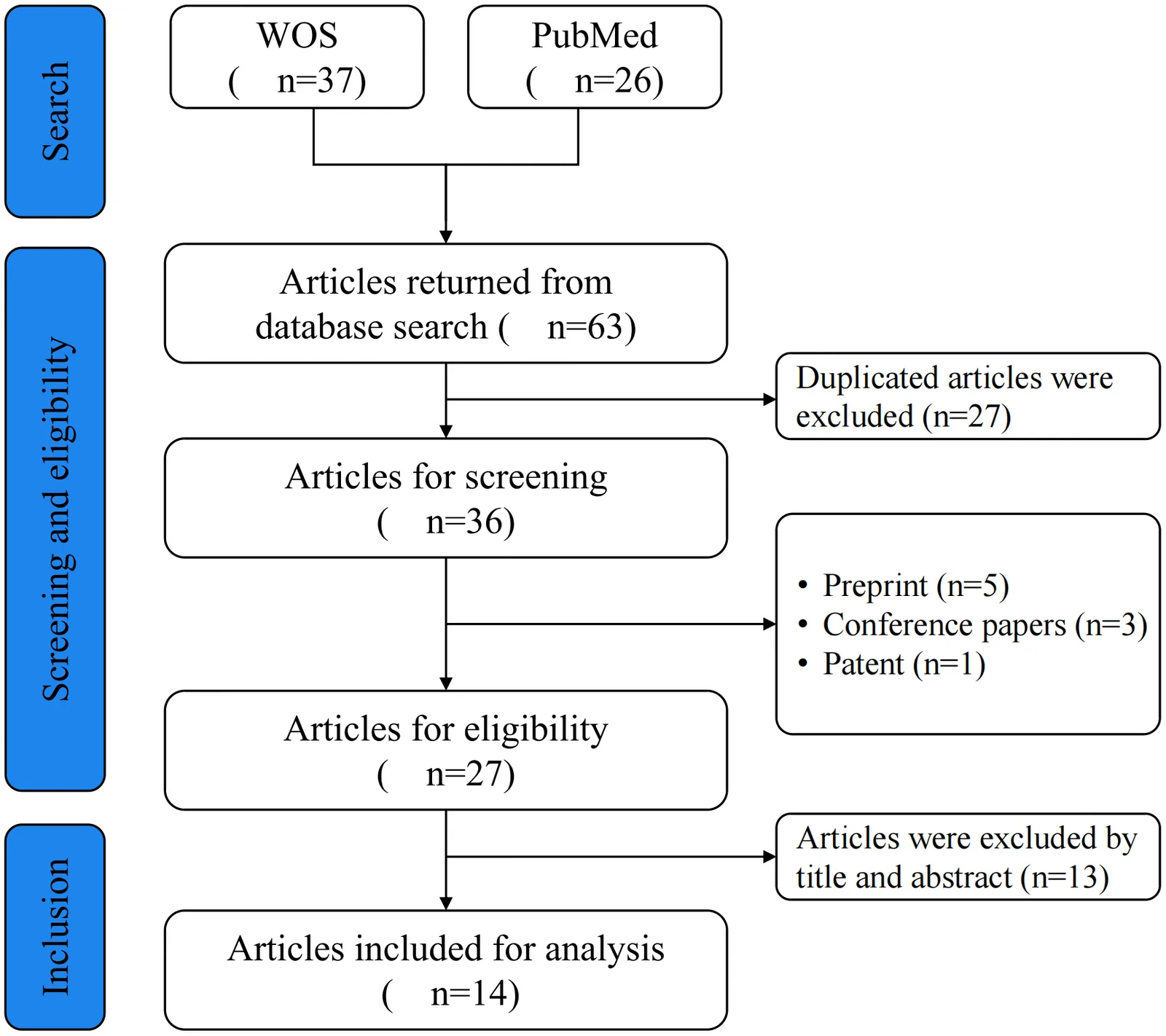
Flow diagram illustrating articles screening and inclusion process.

### Quality assessment of included studies

The quality assessment results for all included studies are summarized in Fig 2. Among the 14 studies included in this review, 10 studies (71.43%) were rated as high quality, 4 studies (28.57%) were rated as moderate quality. No studies were rated as low quality. These results indicate that most studies evaluating LLM performance in the context of the CNMLE and related medical examinations are methodologically robust, and adhered to methodological practices, with relatively clear reporting of data sources, model specifications, and evaluation metrics. However, limitations still existed in areas such as exam year (item 2), data availability (item 6), LLMs prompting strategy disclosure (item 8), and LLMs usage reporting (item 9). Specifically, 4 studies did not specify the exam year used, which may introduce uncertainty in the comparability of LLM performance across different test versions. 9 studies lacked data availability statements, making it difficult for other researchers to reproduce or extend their findings. In addition, 5 studies did not disclose the specific prompting strategies or templates used, which may substantially influence model behavior and evaluation outcomes. In addition, we have summarized detailed LLM usage information in Table 1. This table includes the evaluation date, whether the model was accessed through an API or web interface, the specific model versions or build identifiers, main model parameters (e.g., temperature, top-p, and maximum tokens), and the use of prompting strategies such as standard prompts, chain-of-thought (CoT) reasoning, or retrieval-augmented generation (RAG). The table also indicates whether a predefined prompt template or system prompt was provided.

**Table 1:** Summary of methodological variables across included studies.

Studies	Evaluation date	API vs web UI	Model version	Model parameters	Prompting strategy	Prompt templates
Zong et al. [14]	Not provided	API	GPT-3.5	Not provided	Prompts	Provided
Luo et al. [15]	June 10–30, 2024	Web UI	GPT-3.5, GPT-4, GPT-4o	Temperature: 0.7	Prompts	Not provided
Wu et al. [16]	March 3–9, 2025	Web UI	DeepSeek-R1, GPT-4o	Not provided	Not provided	Not provided
Wang et al. [17]	December 29, 2024	Not provided	GPT-4o, ERNIE	Not provided	Prompts	Provided
Wang et al. [18]	Not provided	Web UI	GPT-4	Not provided	Prompts	Provided
Hong et al. [19]	March 5–10, 2024	Web UI	GPT 3.5, GPT-4	Not provided	Prompts	Provided
Zhang et al. [23]	Not provided	API	GPT-4, ERNIE, DISC-MedLLM	Not provided	Prompts	Provided
Ming et al. [24]	April 24 - May 15, 2023	Web UI	GPT-3.5, GPT-4	Temperature: 0.7	Prompts	Provided
Wu et al. [25]	Not provided	API	GPT-3.5, GPT-4, Baichuan2-7B, Baichuan2-13B, QWEN-72B-Chat	Temperature: 0; top_p: 1; max_tokens: 4096	Prompts+CoT + RAG	Provided
Fang et al. [26]	Not provided	Web UI	GPT-4	Not provided	Prompts	Provided
Tong et al. [27]	May 3–4, 2023	Web UI	GPT-4	Not provided	Prompts	Not provided
Shang et al. [28]	June 12–13, 2023	Web UI	GPT-3.5	Not provided	Not provided	Not provided
Wang et al. [29]	Not provided	Not provided	GPT-3.5	Not provided	Prompts	Not provided
Wang et al. [30]	Feb 14, 2023 and May 14–16, 2023	Web UI	GPT-3.5, GPT-4	Not provided	Prompts	Provided

**Fig 2:**
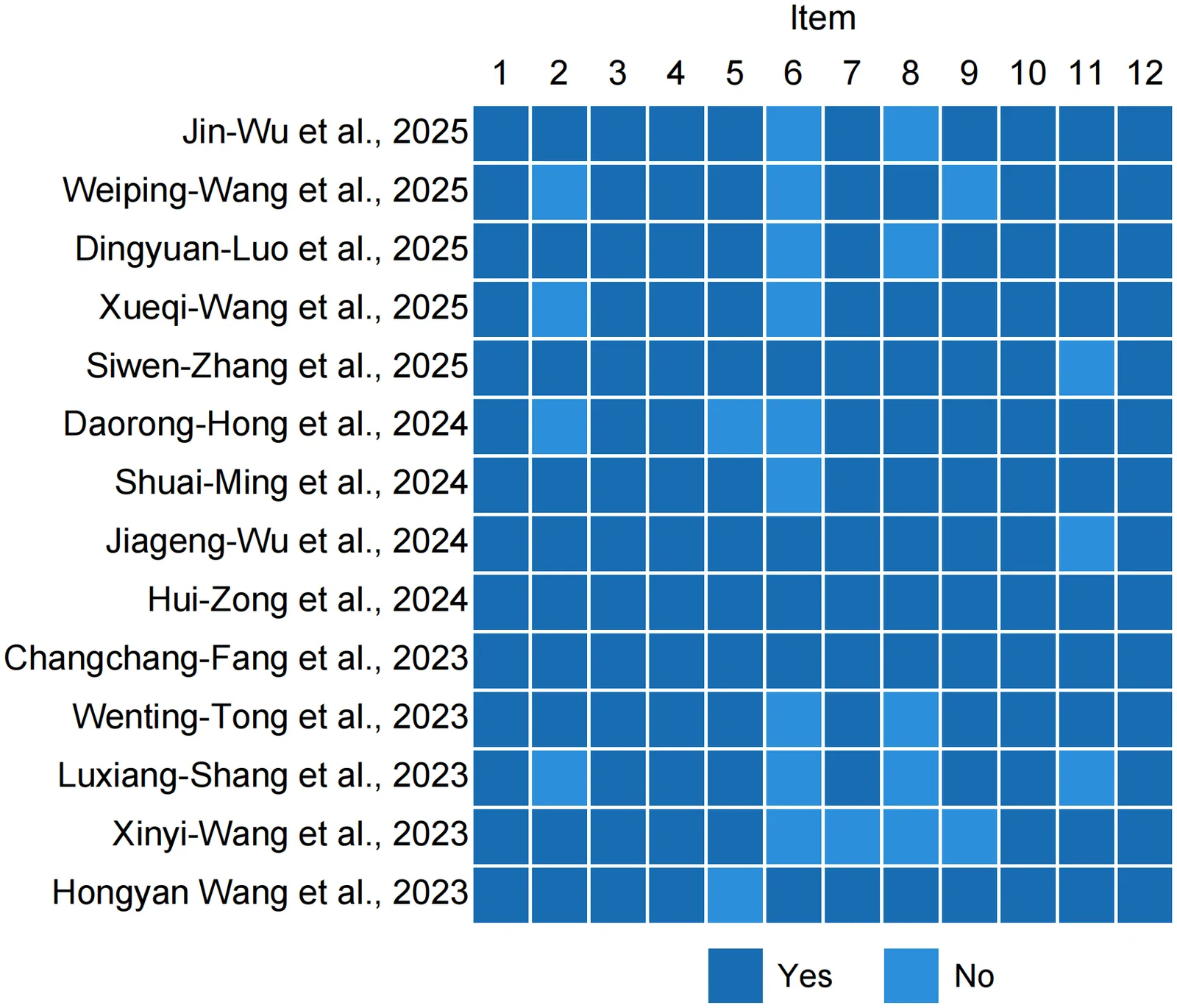
Quality assessment of the included studies based on a 12-item custom checklist.

### Overview of included studies

Fig 3 presents an overall descriptive summary of the included studies and evaluation records across multiple dimensions, including medical exam types, medical exam year, LLMs evaluated, and pass or fail outcomes. As shown in Fig 3A, the CNMLE was the most frequently used exam, with 11 publications and 32 evaluation records. Other exams, such as the Chinese National Pharmacist Licensing Examination (CNPLE) and the Chinese National Nurse Licensing Examination (CNNLE), were each investigated in one publication, contributing 5 records respectively [14]. A few studies also evaluated specialized exams, including Chinese National Health Professional Technical Qualification Examination (Intermediate Level) for Radiation Oncology (CNHPQE-RQ) [17], Chinese Intermediate Professional Technical Qualification Examination for Ultrasound Medicine (CIPTQE-UM) [19], China National Entrance Examination for Postgraduate Clinical Medicine Comprehensive Ability (NEEPM) [30], and Chinese Health Professional Technical Qualification Examination for Critical Care Medicine (CHPTQE-CCM) [18], each contributing 1–2 records.

**Fig 3:**
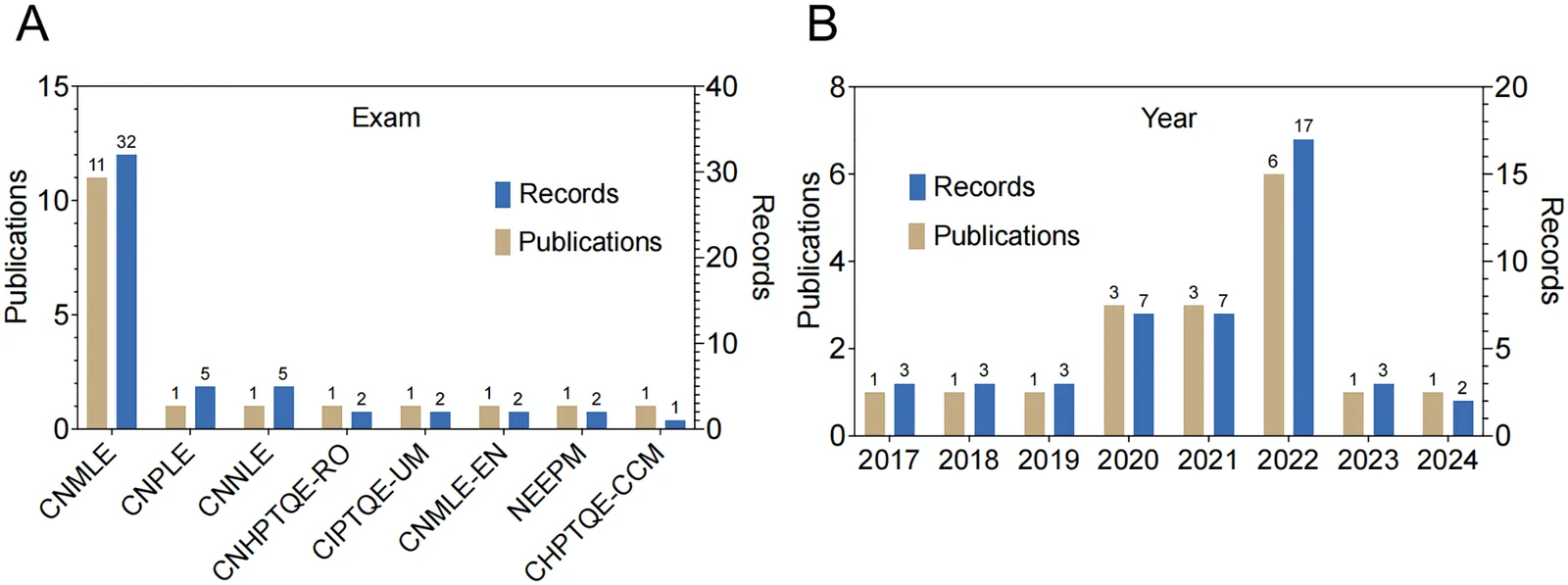
Distribution of the number of publications and records across (A) exam types, (B) exam years. Each bar reflects the frequency of either publications or records reported in the included studies. CNMLE: Chinese National Medical Licensing Examination; CNPLE: Chinese National Pharmacist Licensing Examination; CNNLE: Chinese National Nurse Licensing Examination; CNHPQE-RQ: Chinese National Health Professional Technical Qualification Examination (Intermediate Level) for Radiation Oncology; CIPTQE-UM: Chinese Intermediate Professional Technical Qualification Examination for Ultrasound Medicine; NEEPM: China National Entrance Examination for Postgraduate Clinical Medicine Comprehensive Ability; CHPTQE-CCM: Chinese Health Professional Technical Qualification Examination for Critical Care Medicine. CNMLE-EN: Chinese National Medical Licensing Examination-English Translation Version.

Fig 3B illustrates the distribution of records based on the year of the medical exams used in the included studies. The exam materials spanned from 2017 to 2024, reflecting a broad temporal range of exams utilized for model evaluation. The most frequently used exam years were 2022 (6 publications and 17 records), 2020 (3 publications and 7 records), and 2021 (3 publications and 7 records), suggesting that recent examinations are more commonly selected as benchmarks for evaluating model performance. Earlier exam years such as 2017–2019 were less frequently used. The inclusion of exam questions from 2023 (1 publications and 3 records) and 2024 (1 publication and 2 records) indicates the use of up-to-date exam content, which may better reflect current clinical knowledge requirements and exam standards.

Fig 4 summarizes the types of LLMs used across studies. LLMs are displayed in descending order according to the number of included publications and evaluation records. GPT-3.5 and GPT-4 were the most frequently evaluated models, included in 8 and 9 publications each, and contributing 27 and 13 records, respectively. More recent models such as GPT-4o and open-source Chinese models (e.g., Baichuan2-7B, Baichuan2-13B, Qwen-72B, ERNIE, and DeepSeek-R1) were evaluated in several studies.

**Fig 4:**
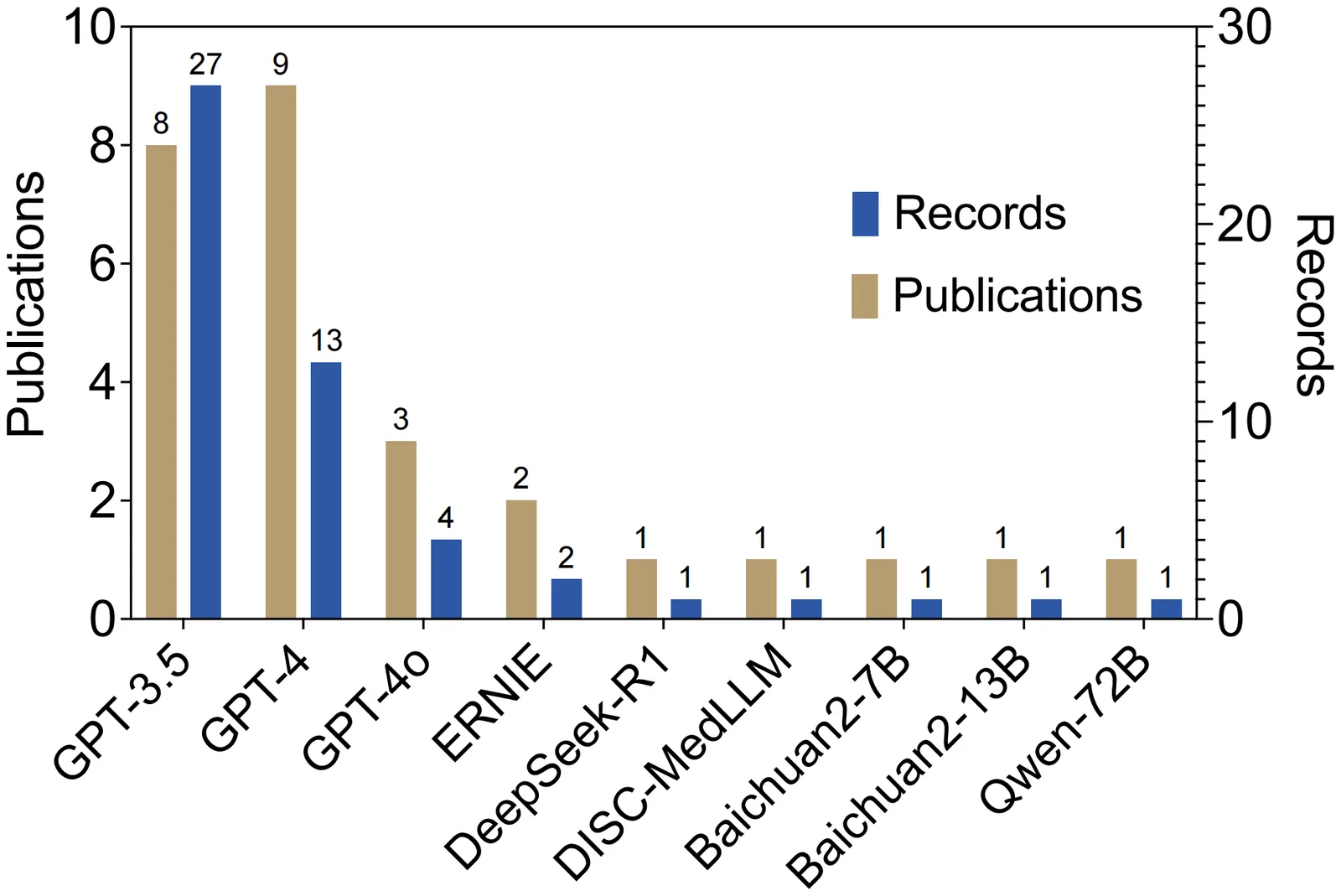
Distribution of the number of publications and records across different large language models.

### The performance of LLMs on CNMLE

Table 2 summarizes the evaluation results of LLMs on the CNMLE which are reported across multiple studies. The exam years covered in these studies range from 2017 to 2024. A clear performance improvement is observed with newer models. Earlier evaluations of GPT-3.5 on CNMLE from 2017 to 2021 consistently showed limited accuracy, typically ranging between 50% and 55%, and failing to meet the passing threshold [14]. In contrast, more recent models such as GPT-4 and GPT-4o demonstrated substantially better performance, with GPT-4o achieving up to 92.00% accuracy on the 2024 CNMLE [16]. Several open-source Chinese LLMs, including DeepSeek-R1, Baichuan2-13B and Qwen-72B, have also been evaluated, with DeepSeek-R1 achieving higher accuracy of 87.20% [16]. The Qwen-72B also achieved a passing-level accuracy of 70.24% [25]. Notably, studies that compared model performance on Chinese versus English versions of the CNMLE found that the same model such as GPT-3.5 or GPT-4 achieved consistently higher accuracy on English-translated questions. For example, GPT-4 achieved 86% accuracy on the English version compared to 84% on the Chinese original, suggesting that current LLMs may still face challenges in handling non-English-language medical content [30].

**Table 2:** Evaluation of large language models (LLMs) on the Chinese National Medical Licensing Examination (CNMLE) and English Translation Version of the CNMLE (CNMLE-EN). “NA” indicates unreported information in the original publication. Note that included studies varied in dataset construction, which may limit direct comparability across studies. For Tong et al., the two GPT-4 records correspond to evaluations conducted on the original Chinese examination and the English-translated version of the same examination, respectively.

Studies	Data resource	Exam year	Number of questions	Models	Number of questions answered correctly	Accuracy	Pass
Zong et al. [14]	CNMLE, Official examination, manually curated	2017	600	GPT-3.5	321	53.50%	No
		2018	600	GPT-3.5	328	54.67%	No
		2019	597	GPT-3.5	306	51.26%	No
		2020	600	GPT-3.5	307	51.17%	No
		2021	600	GPT-3.5	328	54.67%	No
Luo et al. [15]	CNMLE, Complete official examination	2020	600	GPT-3.5	303	50.50%	No
		2020	600	GPT-4	448	74.70%	Yes
		2020	600	GPT-4o	505	84.20%	Yes
		2021	600	GPT-3.5	305	50.80%	No
		2021	600	GPT-4	434	73.20%	Yes
		2021	600	GPT-4o	529	88.20%	Yes
Wu et al. [16]	CNMLE, Complete official examination	2024	600	DeepSeek-R1	523	87.20%	Yes
		2024	600	GPT-4o	552	92.00%	Yes
Zhang et al. [23]	CNMLE, Complete official examination	2023	600	GPT-4	NA	65.20%	Yes
		2023	600	ERNIE	NA	61.70%	Yes
		2023	600	DISC-MedLLM	NA	25.30%	No
Ming et al. [24]	CNMLE, Complete official examination	2022	600	GPT-3.5	324	54%	No
		2022	600	GPT-4	436	72.70%	Yes
Wu et al. [25]	CNMLE, Official examination, manually curated	2022	494	GPT-3.5	NA	51.01%	No
		2022	494	GPT-4	NA	69.23%	Yes
		2022	494	Baichuan2-7B	NA	49.39%	No
		2022	494	Baichuan2-13B	NA	55.87%	No
		2022	494	Qwen-72B	NA	70.24%	Yes
Fang et al. [26]	CNMLE, Complete official examination	2022	600	GPT-4	442	73.67%	Yes
Tong et al. [27]	CNMLE, Official examination, randomly sampled	2022	160 (Chinese)	GPT-4	130	81.25%	Yes
		2022	160 (English-translated)	GPT-4	138	86.25%	Yes
Shang et al. [28]	CNMLE, Online question bank	NA	600	GPT-3.5	340	57.00%	No
Wang et al. [29]	CNMLE, Complete official examination	2020	600	GPT-3.5	282	47.00%	No
		2021	600	GPT-3.5	275	45.80%	No
		2022	600	GPT-3.5	219	36.50%	No
Wang et al. [30]	CNMLE, Official examination, manually curated	2022	100	GPT-3.5	56	56%	NA
		2022	100	GPT-4	84	84%	NA
	CNMLE-EN, Official examination, manually curated	2022	100	GPT-3.5	76	76%	NA
		2022	100	GPT-4	86	86%	NA

In the CNMLE, almost all items are text-based multiple-choice questions. For instance, Wu et al. [16] evaluated 600 questions, all of which were text-based with no visual components. Similarly, Zong et al. [14] analyzed the complete CNMLE question sets from 2017 to 2021, excluding only three items from the 2019 dataset. Across the included studies, most used the full available datasets, while a few selected subsets for analysis. For example, Tong et al. [27] randomly selected 160 questions, and Wu et al. [25] curated 494 multiple-choice questions from the most recent CNMLE held in August 2022 for evaluation. Wang et al. [30] constructed three datasets, each containing 100 manually selected questions. Importantly, the datasets used across studies differed in their representativeness of the actual national licensing examination. While several studies evaluated complete, official CNMLE question sets from specific years, others relied on randomly sampled subsets or manually curated question collections derived from examination materials. These partial or reconstructed datasets may not fully capture the breadth and difficulty distribution of the full CNMLE. In addition, substantial heterogeneity existed across studies in terms of exam year, dataset sampling strategy, prompt engineering approaches, model access methods, and model versions. Therefore, the reported performance metrics should primarily be interpreted as descriptive summaries within individual study contexts rather than strictly comparable benchmarks across studies.

### The performance of LLMs on specialized medical examinations

Table 3 summarizes the evaluation results of LLMs across a range of specialized clinical medical examinations beyond the CNMLE. These include domain-specific qualification assessments such as those for radiation oncology, critical care, ultrasound medicine, pharmacy, nursing, and postgraduate clinical medicine. Among the studies reviewed, the GPT-4 demonstrated the highest model performance with an accuracy of 79.30% (82/100) in NEEPM [30]. In CNHPQE-RQ, GPT-4o achieved an accuracy of 79.30% (895/1128), while ERNIE followed closely with 76.90% (867/1128) [17]. Both records successfully passed the defined passing threshold. Similarly, GPT-4 passed the CHPTQE-CCM with 73.5% accuracy (441/600 questions) [18]. In contrast, performance on the CIPTQE-UM was lower. GPT-3.5 and GPT-4 achieved only 34% and 58% accuracy, respectively, both below the reported pass criteria [19]. Likewise, GPT-3.5 failed to meet passing thresholds across five consecutive years of the CNPLE and CNNLE [14]. It should be noted that the specialized examinations summarized in this section differ substantially in exam purpose, clinical specialty, target population, scoring standards, and dataset construction. Consequently, the reported performance metrics should be interpreted within the context of each individual assessment rather than as directly comparable indicators across examinations.

**Table 3:** The evaluation results of large language models (LLMs) across a range of specialized clinical medical examinations. “NA” indicates unreported information in the original publication.

Studies	Data resource	Exam year	Number of questions	Models	Number of questions answered correctly	Accuracy	Pass
Zong et al. [14]	CNPLE, Official examination, manually curated	2017	459	GPT-3.5	257	55.99%	No
		2018	450	GPT-3.5	223	49.56%	No
		2019	466	GPT-3.5	203	43.56%	No
		2020	458	GPT-3.5	220	48.03%	No
		2021	463	GPT-3.5	222	47.95%	No
	CNNLE, Official examination, manually curated	2017	234	GPT-3.5	138	58.97%	No
		2018	232	GPT-3.5	131	56.47%	No
		2019	238	GPT-3.5	129	54.20%	No
		2020	232	GPT-3.5	107	46.12%	No
		2021	238	GPT-3.5	130	54.62%	No
Wang et al. [17]	CNHPQE-RQ, officially released examination question bank	NA	1128	GPT-4o	895	79.30%	Yes
		NA	1128	ERNIE	867	76.90%	Yes
Wang et al. [18]	CHPTQE-CCM, Official question bank, stratified random sampling	NA	600	GPT-4	441	73.50%	Yes
Hong et al. [19]	CIPTQE-UM, Question bank, random sampling	NA	100	GPT-3.5	34	34%	No
		NA	100	GPT-4	58	58%	No
Wang et al. [30]	NEEPM, Official examination, manually curated	2022	100	GPT-3.5	62	62%	NA
		2022	100	GPT-4	82	82%	NA

### Comparison of pass rates across LLMs

Among all 51 evaluation records, the most frequently tested models were GPT-3.5 (n = 27) and GPT-4 (n = 13). For GPT-3.5, 24 records that reported pass or fail outcomes, of which no one met the passing threshold. In contrast, among the 10 GPT-4 records with reported pass or fail outcomes, 9 achieved passing-level performance. As an exploratory, hypothesis-generating analysis, we used Fisher’s exact test (two-sided) to compare observed pass rate patterns across records. The difference reached statistically significant (P < 0.001). However, this result should be interpreted cautiously because the included studies differed substantially in exam year, dataset construction, sampling strategies, prompt engineering methods, model versions, and evaluation settings, which may limit direct comparability across records. This result should be interpreted strictly as descriptive and should not be taken as definitive evidence of model superiority.

## Discussion

### Principal findings

This scoping review comprehensively synthesizes the current evidence on the performance of LLMs in the CNMLE and related standardized medical assessments. We identified 14 peer-reviewed studies comprising 51 evaluation records. Our results suggest an apparent trend of higher reported accuracy in newer LLMs such as GPT-4, GPT-4o and DeepSeek, which demonstrate substantial improvements over earlier models like GPT-3.5, with many achieving scores above the official passing threshold. However, this pattern should be interpreted as descriptive rather than as direct evidence of temporal model improvement. An exploratory statistical comparison is consistent with this pattern, with GPT-4 showing a higher reported pass rate than GPT-3.5. Nevertheless, this comparison is descriptive and does not establish definitive model superiority given the heterogeneity of evaluation contexts. In addition to CNMLE, LLMs have also been tested across various domain-specific medical exams, including critical care, radiation oncology, and ultrasound medicine. Nevertheless, these findings should be interpreted cautiously, as strong performance on standardized examinations does not necessarily indicate real-world clinical competence. This review provides the first structured overview of these developments and offers a critical assessment of their methodological quality and limitations.

From a digital health perspective, these findings have broader implications for health workforce education, assessment standardization, and AI governance. The increasing performance of LLMs on national licensing examinations suggests potential utility in supporting medical training and formative assessment, particularly in settings with limited educational resources. However, this also raises important concerns regarding examination integrity, regulatory oversight, and equitable access to high-quality medical education tools. In low- and middle-income country contexts, where educational resources may be unevenly distributed, LLMs could help reduce training gaps, but may also exacerbate disparities if access and model quality are not carefully regulated. Therefore, the integration of LLMs into medical education systems should be guided by transparent governance frameworks and alignment with national licensing authorities.

### Current research status and trends

The application of LLMs in medical licensing exams represents a growing intersection between artificial intelligence and medical education [31–33]. Recent reviews and surveys have further highlighted the rapidly expanding role of LLMs across diverse medical domains, including clinical decision support, medical education, report generation, and biomedical knowledge reasoning [34,35]. In addition, emerging multimodal medical LLMs capable of integrating imaging and textual information have demonstrated substantial potential for future intelligent healthcare systems and pandemic response scenarios [36]. These developments provide important context for understanding the increasing interest in evaluating LLMs using standardized medical licensing examinations. There has been a clear increase in the number and scope of studies evaluating LLMs on CNMLE and related exams. Initially, most studies focused narrowly on the CNMLE, using a small set of test questions and early LLMs such as GPT-3.5. However, this landscape is evolving in three notable ways.

First, the range of medical examinations assessed has broadened significantly. While CNMLE remains the most studied benchmark, recent evaluations have incorporated a variety of domain-specific exams, including specialist exams in radiation oncology, critical care, and ultrasound medicine. This diversification reflects an increasing interest in exploring the generalizability of LLMs across different clinical disciplines and licensing systems. Second, there is a growing tendency to evaluate LLMs on more recent exam content. Exams from the years 2022–2024 are being used with increasing frequency, likely to ensure that test items reflect the most current medical knowledge, clinical practices, and regulatory standards. Third, the range of evaluated LLMs is becoming increasingly diverse. Earlier studies focused primarily on proprietary models like GPT-3.5, but more recent work has incorporated advanced versions such as GPT-4o and a growing number of high-performing Chinese open-source models, including DeepSeek, Qwen, and Baichuan2. These models demonstrate competitive performance and signal a move toward localized, language-specific model development and evaluation. This is also consistent with recent efforts to construct large-scale Chinese medical benchmark datasets for systematic evaluation of LLMs. For example, the CMExam benchmark [37] and the CNMLEQA dataset [38] introduced by recent studies have provided valuable resources for assessing medical reasoning, factual knowledge, and multilingual adaptability in Chinese medical contexts.

These trends highlight the growing depth and complexity of research on LLMs in Chinese medical education, paving the way for more rigorous, standardized, and clinically meaningful evaluations in the future.

### Challenges of using LLMs in CNMLE

Despite promising performance in recent models, multiple challenges remain. First, methodological inconsistencies exist across studies. Our quality assessment revealed that some studies did not clearly report exam years, model prompting strategies, or model evaluation details, which limits reproducibility and comparability. Second, many evaluations rely on filtered question sets, excluding multimedia items like images or tables. While necessary for text-only models, this does not fully represent real-world exam conditions and may overestimate performance. Third, studies that compared English and Chinese versions of the CNMLE consistently found better performance in English, even when using the same model. This suggests that current LLMs may still struggle with medical context in Chinese language, especially in specialized terminology and reasoning tasks. Fourth, previous studies have demonstrated that high benchmark scores may partially reflect memorization of training data patterns rather than genuine clinical reasoning capability, potentially leading to overestimation of real-world performance [39]. Some CNMLE questions and related preparation materials may be publicly available online, the potential influence of benchmark contamination or training data leakage cannot be fully excluded. Therefore, strong benchmark performance should be interpreted cautiously and may not necessarily reflect genuine medical reasoning ability or medical competence. Finally, the lack of standardized benchmark datasets and evaluation protocols continues to limit cross-study synthesis. In addition, some studies do not disclose the evaluation dataset or detailed testing results.

### Future direction of LLMs application in CNMLE

To advance the field, several directions should be prioritized. First, the development of a standardized, open-access CNMLE benchmark dataset would significantly enhance comparability and reproducibility [37,40]. Such a benchmark should include a representative sample of question types and modalities (e.g., text, image, table), aligned with official licensing exam standards. This is particularly important given the increasing development of multimodal medical AI systems for comprehensive medical assessment [41]. Second, future studies should more rigorously document prompt templates, LLM versions and evaluation date, usage details (e.g., API vs. web UI), and evaluation metrics. Third, the multilingual and cultural adaptability of LLMs needs to be improved [42]. As demonstrated by the performance gaps between English and Chinese versions of the same exam, there is a need to train or fine-tune models using large-scale, high-quality Chinese medical corpora. Integration with retrieval-augmented generation pipelines, medical ontologies and knowledge graph frameworks may further improve reasoning quality in factual consistency, faithfulness, and patient instruction generation [43–45]. Fourth, while achieving high scores on licensing exams demonstrates domain knowledge, this does not directly equate to clinical competency, safe medical practice, or readiness for autonomous deployment in healthcare settings. Future research must investigate the transferability of this exam performance to real-world clinical utility. Therefore, real-world deployment scenarios such as medical student training and clinical decision support could serve as testing grounds for longitudinal evaluation of LLM utility [46]. Moreover, responsible usage, transparency, and alignment with medical ethics should remain central pillars in this domain.

### Limitations

This study has several limitations. First, the literature search was primarily conducted using PubMed and WOS, which may have excluded relevant studies indexed in other databases. However, supplementary searches in Chinese literature databases, such as CNKI and WanFang, as well as IEEE Xplore and Scopus database yielded no additional eligible records, suggesting that the included studies likely represent the vast majority of relevant publications in this field. Second, the current evaluation of LLMs has been largely limited to language models. While this reflects the capabilities of most mainstream LLMs at the time of study, it overlooks the growing relevance of multimodal models. Moreover, strong performance on standardized examinations should not be interpreted as equivalent to genuine clinical reasoning ability or readiness for real-world medical practice, as examination-based benchmarks capture only a limited aspect of clinical competence. The included studies also differed substantially in datasets, examination years, prompt engineering approaches, and evaluation settings. Future research should consider incorporating LLMs with visual understanding capabilities, as some medical examination questions include images, tables, and other non-textual content that are beyond the scope of current text-only models. In addition, because most of medical examination questions may have been publicly available online, the potential influence of benchmark contamination or memorization effects cannot be fully excluded. Future evaluations should therefore incorporate stricter contamination control and external validation strategies.

## Conclusion

This study provides a comprehensive synthesis of current research evaluating LLMs on the CNMLE and related medical exams. The increasing use of diverse medical exam types and open-source LLMs reflect rapid advances and growing interest in this field. Our findings highlight a growing body of evidence supporting the improving performance of LLMs, particularly models like GPT-4o and DeepSeek-R1 on complex, real-world, non-English medical examinations. Despite these advancements, significant methodological variability remains. Heterogeneity in dataset availability, prompt design, LLMs versions, and reporting of pass or fail outcomes limit cross-study comparability and generalizability. Future research should develop standardized benchmarks, incorporate multimodal LLMs, and establish transparent, reproducible evaluation protocols.

## Supporting information

S1 PRISMA Checklist Preferred Reporting Items for Systematic reviews and Meta-Analyses extension for Scoping Reviews (PRISMA-ScR) Checklist. (DOCX)

S1 Appendix Search strategies and search results across different databases. Database searches conducted on June 11, 2025.
(DOCX)

S2 Appendix Checklist used to assess the reporting quality and methodological transparency of studies evaluating large language model (LLM) on medical examinations. The checklist comprises 12 items across three domains: Dataset Characteristics, LLM Testing Setup, and Evaluation Methods. For each item, there are two possible responses: Y for Yes and N for No. A quality score is calculated for each study based on the number of “Yes” responses, with 1 point awarded per “Yes”. Studies are categorized based on total score: High quality (≥10 points), Moderate quality (8–9 points), Low quality (<8 points). This scoring framework allows for a structured comparison of study rigor and completeness.
(DOCX)

## References

[1] Sandmann S, Hegselmann S, Fujarski M, Bickmann L, Wild B, Eils R. Benchmark evaluation of DeepSeek large language models in clinical decision-making. Nat Med. 2025. doi: 10.1038/s41591-025-03727-2 40267970 PMC12353792

[2] Tordjman M, Liu Z, Yuce M, Fauveau V, Mei Y, Hadjadj J. Comparative benchmarking of the DeepSeek large language model on medical tasks and clinical reasoning. Nat Med. 2025. doi: 10.1038/s41591-025-03726-3 40267969

[3] Mirza FN, Tang OY, Connolly ID, Abdulrazeq HA, Lim RK, Roye GD, et al. Using ChatGPT to facilitate truly informed medical consent. NEJM AI. 2024;1(2). doi: 10.1056/aics2300145

[4] Yang A, Li A, Yang B, Zhang B, Hui B, Zheng B. Qwen3 technical report. 2025. https://arxiv.org/abs/250509388

[5] Zong H, Wu R, Cha J, Feng W, Wu E, Li J, et al. Advancing Chinese biomedical text mining with community challenges. J Biomed Inform. 2024;157:104716. doi: 10.1016/j.jbi.2024.104716 39197732

[6] Ouyang D, Hogan J. We need more randomized clinical trials of AI. NEJM AI. 2024;1(11):AIe2400881. doi: 10.1056/AIe2400881

[7] Wan P, Huang Z, Tang W, Nie Y, Pei D, Deng S, et al. Outpatient reception via collaboration between nurses and a large language model: A randomized controlled trial. Nat Med. 2024;30(10):2878–85. doi: 10.1038/s41591-024-03148-7 39009780

[8] Drazen JM, Haug CJ. Trials of AI interventions must be preregistered. NEJM AI. 2024;1(4). doi: 10.1056/aie2400146

[9] Pati A, Addula SR, Panigrahi A, Sahu B, Nayak DSK, Dash M. Artificial intelligence in improving disease diagnosis: A case study of cardiovascular disease prediction. Artificial Intelligence in Medicine and Healthcare. CRC Press; 2025. pp. 24–49.

[10] Liu M, Okuhara T, Chang X, Shirabe R, Nishiie Y, Okada H, et al. Performance of ChatGPT Across Different Versions in Medical Licensing Examinations Worldwide: Systematic Review and Meta-Analysis. J Med Internet Res. 2024;26:e60807. doi: 10.2196/60807 39052324 PMC11310649

[11] Raji ID, Daneshjou R, Alsentzer E. It’s time to bench the medical exam benchmark. NEJM AI. 2025;2(2). doi: 10.1056/aie2401235

[12] Zong H, Wu R, Cha J, Wang J, Wu E, Li J, et al. Large language models in worldwide medical exams: Platform development and comprehensive analysis. J Med Internet Res. 2024;26:e66114. doi: 10.2196/66114 39729356 PMC11724220

[13] Wang X. Experiences, challenges, and prospects of National Medical Licensing Examination in China. BMC Med Educ. 2022;22(1):349. doi: 10.1186/s12909-022-03385-9 35527240 PMC9079212

[14] Zong H, Li J, Wu E, Wu R, Lu J, Shen B. Performance of ChatGPT on Chinese national medical licensing examinations: a five-year examination evaluation study for physicians, pharmacists and nurses. BMC Med Educ. 2024;24(1):143. doi: 10.1186/s12909-024-05125-7 38355517 PMC10868058

[15] Luo D, Liu M, Yu R, Liu Y, Jiang W, Fan Q, et al. Evaluating the performance of GPT-3.5, GPT-4, and GPT-4o in the Chinese National Medical Licensing Examination. Sci Rep. 2025;15(1):14119. doi: 10.1038/s41598-025-98949-2 40269046 PMC12018924

[16] Wu J, Wang Z, Qin Y. Performance of DeepSeek-R1 and ChatGPT-4o on the Chinese National Medical Licensing Examination: A Comparative Study. J Med Syst. 2025;49(1):74. doi: 10.1007/s10916-025-02213-z 40459679

[17] Wang W, Fu J, Zhang Y, Hu K. A comparative analysis of GPT-4o and ERNIE Bot in a Chinese radiation oncology exam. J Cancer Educ. 2025. doi: 10.1007/s13187-025-02652-9 40418520

[18] Wang X, Tang J, Feng Y, Tang C, Wang X. Can ChatGPT-4 perform as a competent physician based on the Chinese critical care examination? J Crit Care. 2025;86:155010. doi: 10.1016/j.jcrc.2024.155010 40023616

[19] Hong D-R, Huang C-Y. The performance of AI in medical examinations: An exploration of ChatGPT in ultrasound medical education. Front Med (Lausanne). 2024;11:1472006. doi: 10.3389/fmed.2024.1472006 39564507 PMC11573551

[20] Tricco AC, Lillie E, Zarin W, O’Brien KK, Colquhoun H, Levac D, et al. PRISMA extension for scoping reviews (PRISMA-ScR): Checklist and explanation. Ann Intern Med. 2018;169(7):467–73. doi: 10.7326/M18-0850 30178033

[21] Jin Q, Leaman R, Lu Z. PubMed and beyond: Biomedical literature search in the age of artificial intelligence. EBioMedicine. 2024;100:104988. doi: 10.1016/j.ebiom.2024.104988 38306900 PMC10850402

[22] Mongeon P, Paul-Hus A. The journal coverage of Web of Science and Scopus: A comparative analysis. Scientometrics. 2015;106(1):213–28. doi: 10.1007/s11192-015-1765-5

[23] Zhang S, Chu Q, Li Y, Liu J, Wang J, Yan C, et al. Evaluation of large language models under different training background in Chinese medical examination: A comparative study. Front Artif Intell. 2024;7:1442975. doi: 10.3389/frai.2024.1442975 39697797 PMC11652508

[24] Ming S, Guo Q, Cheng W, Lei B. Influence of model evolution and system roles on ChatGPT’s performance in Chinese Medical Licensing Exams: Comparative Study. JMIR Med Educ. 2024;10:e52784. doi: 10.2196/52784 39140269 PMC11336778

[25] Wu J, Wu X, Qiu Z, Li M, Lin S, Zhang Y, et al. Large language models leverage external knowledge to extend clinical insight beyond language boundaries. J Am Med Inform Assoc. 2024;31(9):2054–64. doi: 10.1093/jamia/ocae079 38684792 PMC11339525

[26] Fang C, Wu Y, Fu W, Ling J, Wang Y, Liu X, et al. How does ChatGPT-4 preform on non-English national medical licensing examination? An evaluation in Chinese language. PLOS Digit Health. 2023;2(12):e0000397. doi: 10.1371/journal.pdig.0000397 38039286 PMC10691691

[27] Tong W, Guan Y, Chen J, Huang X, Zhong Y, Zhang C, et al. Artificial intelligence in global health equity: an evaluation and discussion on the application of ChatGPT, in the Chinese National Medical Licensing Examination. Front Med (Lausanne). 2023;10:1237432. doi: 10.3389/fmed.2023.1237432 38020160 PMC10656681

[28] Shang L, Xue M, Hou Y, Tang B. Can ChatGPT pass China’s national medical licensing examination? Asian J Surg. 2023;46(12):6112–3. doi: 10.1016/j.asjsur.2023.09.089 37775381

[29] Wang X, Gong Z, Wang G, Jia J, Xu Y, Zhao J. ChatGPT performs on the Chinese national medical licensing examination. J Med Syst. 2023;47(1):86. doi: 10.1007/s10916-023-01961-0 37581690

[30] Wang H, Wu W, Dou Z, He L, Yang L. Performance and exploration of ChatGPT in medical examination, records and education in Chinese: Pave the way for medical AI. Int J Med Inform. 2023;177:105173. doi: 10.1016/j.ijmedinf.2023.105173 37549499

[31] Artsi Y, Sorin V, Konen E, Glicksberg BS, Nadkarni G, Klang E. Large language models for generating medical examinations: Systematic review. BMC Med Educ. 2024;24(1):354. doi: 10.1186/s12909-024-05239-y 38553693 PMC10981304

[32] Singhal K, Tu T, Gottweis J, Sayres R, Wulczyn E, Amin M, et al. Toward expert-level medical question answering with large language models. Nat Med. 2025;31(3):943–50. doi: 10.1038/s41591-024-03423-7 39779926 PMC11922739

[33] Kung TH, Cheatham M, Medenilla A, Sillos C, De Leon L, Elepaño C, et al. Performance of ChatGPT on USMLE: Potential for AI-assisted medical education using large language models. PLOS Digit Health. 2023;2(2):e0000198. doi: 10.1371/journal.pdig.0000198 36812645 PMC9931230

[34] Zhou H, Liu F, Gu B, Zou X, Huang J, Wu J, et al. A survey of large language models in medicine: Progress, application, and challenge. arXiv preprint arXiv:231105112. 2023.

[35] Liu F, Zhou H, Gu B, Zou X, Huang J, Wu J, et al. Application of large language models in medicine. Nat Rev Bioeng. 2025;3(6):445–64. doi: 10.1038/s44222-025-00279-5

[36] Liu F, Zhu T, Wu X, Yang B, You C, Wang C, et al. A medical multimodal large language model for future pandemics. NPJ Digit Med. 2023;6(1):226. doi: 10.1038/s41746-023-00952-2 38042919 PMC10693607

[37] Liu J, Zhou P, Hua Y, Chong D, Tian Z, Liu A. Benchmarking large language models on CMExam: A comprehensive Chinese Medical Exam Dataset. Adv Neural Inform Process Syst. 2023;36:52430–52.

[38] Zong H, Cha J, Wang J, Song Y, Zhao Y, Shi M, et al. A dataset for evaluating large language models on Chinese National Medical Licensing Examinations. Scientific Data. 2026. doi: 10.1038/s41597-026-07261-9 41998016 PMC13272756

[39] You C, Dai H, Min Y, Sekhon JS, Joshi S, Duncan JS. Uncovering memorization effect in the presence of spurious correlations. Nat Commun. 2025;16(1):5424. doi: 10.1038/s41467-025-61531-5 40593835 PMC12216586

[40] Liu M, Hu W, Ding J, Xu J, Li X, Zhu L, et al. MedBench: A comprehensive, standardized, and reliable benchmarking system for evaluating Chinese Medical Large Language Models. Big Data Min Anal. 2024;7(4):1116–28. doi: 10.26599/bdma.2024.9020044

[41] Henao JAG, Depotter A, Bower DV, Bajercius H, Todorova PT, Saint-James H, et al. A multiclass radiomics method-based WHO Severity Scale for improving COVID-19 patient assessment and disease characterization from CT scans. Invest Radiol. 2023;58(12):882–93. doi: 10.1097/RLI.0000000000001005 37493348 PMC10662611

[42] Qin L, Chen Q, Zhou Y, Chen Z, Li Y, Liao L, et al. A survey of multilingual large language models. Patterns (N Y). 2025;6(1):101118. doi: 10.1016/j.patter.2024.101118 39896256 PMC11783891

[43] Amugongo LM, Mascheroni P, Brooks S, Doering S, Seidel J. Retrieval augmented generation for large language models in healthcare: A systematic review. PLOS Digit Health. 2025;4(6):e0000877. doi: 10.1371/journal.pdig.0000877 40498738 PMC12157099

[44] Liu F, You C, Wu X, Ge S, Wang S, Sun X. Auto-encoding knowledge graph for unsupervised medical report generation. Proceedings of the 35th International Conference on Neural Information Processing Systems. Curran Associates Inc.; 2021. pp. Article 1244.

[45] Liu F, Yang B, You C, Wu X, Ge S, Liu Z, et al. Retrieve, reason, and refine: generating accurate and faithful patient instructions. Proceedings of the 36th International Conference on Neural Information Processing Systems. New Orleans, LA, USA: Curran Associates Inc.; 2022. pp. Article 1370.

[46] Li J, Zong H, Wu E, Wu R, Peng Z, Zhao J. Exploring the potential of artificial intelligence to enhance the writing of english academic papers by non-native english-speaking medical students - the educational application of ChatGPT. BMC Med Educ. 2024;24(1):736. doi: 10.1186/s12909-024-05738-y 38982429 PMC11232216

