# Supplementary material for: Performance of large language models in the Chinese National Medical Licensing Examinations and Beyond: Scoping review
Source: PLOS Digit Health. 2026 Oct 9;5(10):e0001773. doi: 10.1371/journal.pdig.0001773 (PMC13652241; doi:10.1371/journal.pdig.0001773)
Supplement: Supplementary file 2 [file pdig.0001773.s002.docx]

**Table S1.** Search strategies and search results across different databases. Database searches conducted on June 11, 2025.

| **Databases** | **Search formulas** | **Search results** | **Total studies** |
| --- | --- | --- | --- |
| PubMed | (("large language model*"[TIAB] OR "chatgpt*"[TIAB] OR "gpt3.5*"[TIAB] OR "gpt4*"[TIAB] OR "bard*"[TIAB] OR "gemini*"[TIAB] OR "llama*"[TIAB] OR "claude*"[TIAB] OR "ernie*"[TIAB] OR "qwen*"[TIAB] OR "deepseek*"[TIAB]) AND ("medical licensing exam*"[TIAB] OR "physician licensing exam*"[TIAB] OR "medical exam*"[TIAB] OR "physician exam*"[TIAB] OR "NMLE"[TIAB]) AND ("China"[TIAB] OR "Chinese"[TIAB])) | 26 | 63 |
| Web of Science | TS = ("large language model*" OR "chatgpt*" OR "gpt3.5*" OR "gpt4*" OR "bard*" OR "gemini*" OR "llama*" OR "claude*" OR "ernie*" OR "qwen*" OR "deepseek*") AND TS = ("medical licensing exam*" OR "physician licensing exam*" OR "medical exam*" OR "physician exam*" OR "NMLE") AND TS = ("China" OR "Chinese") | 37 |  |
