# Supplementary material for: Performance of large language models in the Chinese National Medical Licensing Examinations and Beyond: Scoping review
Source: PLOS Digit Health. 2026 Oct 9;5(10):e0001773. doi: 10.1371/journal.pdig.0001773 (PMC13652241; doi:10.1371/journal.pdig.0001773)
Supplement: Supplementary file 3 [file pdig.0001773.s003.docx]

**Table S1**. Checklist used to assess the reporting quality and methodological transparency of studies evaluating large language model (LLM) on medical examinations. The checklist comprises 12 items across three domains: Dataset Characteristics, LLM Testing Setup, and Evaluation Methods. For each item, there are two possible responses: Y for Yes and N for No. A quality score is calculated for each study based on the number of “Yes” responses, with 1 point awarded per "Yes". Studies are categorized based on total score: High quality (≥10 points), Moderate quality (8-9 points), Low quality (<8 points). This scoring framework allows for a structured comparison of study rigor and completeness.

| Section | Item | Checklist item |
| --- | --- | --- |
| Dataset Characteristics | 1 | Does the study report the source of the exam? (e.g., CNMLE 2022) |
|  | 2 | Does the study specify the year of the exam? |
|  | 3 | Does the study describe the number of questions used? |
|  | 4 | Does the study clearly state whether any filters were applied to the original question set? (e.g., excludes image-based questions) |
|  | 5 | Does the study define or reference a passing criterion? (e.g., 360/600 official threshold) |
|  | 6 | Does the study indicate whether the dataset is publicly available or accessible? |
| LLM Testing Setup | 7 | Does the study clearly report the specific LLM version? |
|  | 8 | Does the study provide the prompt or examples used in the evaluation? |
|  | 9 | Does the study describe the testing method? (e.g., via API, web interface) |
| Evaluation Methods | 10 | Does the study report evaluation metrics? (e.g., accuracy) |
|  | 11 | Does the study use any statistical methods to compare performance? |
|  | 12 | Does the study state whether the LLM passes the exam based on defined criteria? |
